# The Relationship Between Watching Mukbang (Eating Show), Eating Behaviors, and Anthropometric Parameters in Iranian Female Students

**DOI:** 10.34172/jrhs.2023.109

**Published:** 2023-03-30

**Authors:** Fatemeh Manafi Anari, Shahryar Eghtesadi

**Affiliations:** ^1^Department of Nutrition, Science and Research Branch, Islamic Azad University, Tehran, Iran

**Keywords:** Mukbang, Eating show, Eating behaviors, Anthropometric parameters, Students

## Abstract

**Background:** With the increasing watching of programs such as Mukbang, the study of eating behaviors and anthropometric parameters and their relationship with Mukbang should be considered to prevent chronic diseases and eating disorders. This study investigated the relationship between watching Mukbang with eating behaviors and anthropometric parameters in female students at Islamic Azad University, Science and Research Branch, Tehran, Iran.

**Study Design:** A cross-sectional study.

**Methods:** In this study, 114 female students aged 18 to 31 years were selected using simple random sampling. General information, watching Mukbang status, and the Dutch Eating Behavior Questionnaire (DEBQ) were administered to the participants online.

**Results: ** The prevalence of Mukbang watching in female students of the present study was 60.5%. Furthermore, there was no significant relationship between the frequency of Mukbang watching and emotional or restrained eating behavior (*P* > 0.05), while there was a significant relationship between external eating behavior and the frequency of watching Mukbang (*P*=0.0001). It was found that with increasing watching time, external eating also increased. According to the results of logistic regression analysis, the external eating chance was 27% lower in students who watch Mukbang rarely than those who have never watched such a show. However, this chance was 0.31 and 9.58 times higher in students who occasionally and always watch Mukbang, respectively.

**Conclusion:** There is a significant relationship between external eating behaviors and watching Mukbang.

## Background

 Nowadays, various online activities are on the rise, an example of which is watching others while eating, called Mukbang, which is a South Korean term consisting of “meokneun” (eating) and “bangsong” (broadcast). Mukbang was initially broadcasted online on the Afreeca TV channel in South Korea in 2008, and it expanded around the world in 2015.^1^ Mukbang is a live or prerecorded program in which the host consumes an extraordinarily copious amount of food while interacting with their audience.^2^ Critics believe that Mukbang can increase overeating. Accordingly, the Ministry of Health and Welfare of South Korea formulated a Mukbang monitoring system in 2018 to prevent the prevalence of obesity.^3^

 Many studies have concluded that Mukbang watching can have negative effects on spectators by increasing food consumption due to social comparison or imitation, altering spectators’ understanding of food consumption and eating behaviors through modeling poor behaviors and promoting obesity and various eating disorders through the positive demonstration of showing gluttony.^4-7^ Mukbang may also be watched more frequently by those who show symptoms of eating disorders.^8^

 The term eating behavior can include food choices, eating practices and habits, dieting, and eating disorders involving risk factors such as obesity.^9^ Obesity is characterized by three behavioral and psychological theories, namely Externality theory,^10^ Psychosomatic theory,^11^ and Restrained Eating theory,^12^ each of which describes one’s behavior toward eating. Accordingly, eating behaviors include emotional eating, external eating, and restrained eating.

 The concept of emotional eating suggests the desire to eat in reaction to negative feelings, stemming from the psychosomatic theory. According to this theory, obesity results from a person’s encounter with unfavorable situations and stimuli such as depression and anxiety. External eating refers to eating in reaction to an external stimulus related to food (food view or smell) independent of the inner state of starvation or satiation, which is derived from the externality theory.^13^ Restrained eating reflects a degree of informed restriction derived from restraint. According to this theory, calorie consumption is considerably reduced in a person. In most cases, this restriction is associated with overeating periods.^14^

 Various components of eating behaviors (i.e., emotional eating, external eating, and restrained eating) were found to be correlated with body mass index (BMI) levels. For instance, the components of external eating and restrained eating can result in overweight and obesity through overeating.^13,15^

 In early adulthood, students typically exhibit undesirable eating habits and can have a poor diet as they often eat outside the home, have erratic meals,^16^ possess a small budget and wrong nutrition knowledge, and tend to look excessively beautiful.^17^ Additionally, a survey on using smartphones conducted in South Korea indicated that 25.9% of students in this country were exposed to the risk of over-dependence on smartphones compared to 18.1% of adults. Thus, students can specifically become susceptible to the detrimental consequences of watching Mukbang as the content available through mobile phones is still on the rise rapidly.^18^

 To the best of our knowledge, there has not been a documented study in this field in Iran yet. Hence, it is necessary to focus on studies concerning eating behaviors, anthropometric parameters, and their association with such programs as Mukbang to prevent related chronic diseases and eating disorders and to identify harmful habits to provide solutions for their removal or replacement with acceptable eating behaviors in society. This study, therefore, aimed to investigate the association between watching Mukbang with eating behaviors and anthropometric parameters among Iranian female students.

## Methods

###  Study design and participant

 In the present cross-sectional research, the target population consisted of female students who were studying at the Islamic Azad University, Science and Research Branch, Tehran, Iran, in January 2022. A sample size of 120 students was determined using a related statistical formula and Mukbang-watching frequency.^18^ A final sample of 114 participants aged 18 to 31 years remained after excluding unusual cases with anomaly index, people who had any chronic diseases or conditions that affect eating behaviors and weight status, those who took any supplements or drugs that affect weight status, and those who followed special diets. Samples were chosen by a simple random sampling method. A Google form questionnaire was distributed using email contacts and social networking sites such as Telegram. The aim and procedure of the study were fully explained to the participants, and then their informed consent forms were collected to observe ethical principles. All the students were ensured confidentiality of their information. This study received an ethics code approved by the Islamic Azad University, Science and Research Branch.

###  Data collection

 The research tool consisted of three questionnaires. In the first part, the required data on the age, educational level, and anthropometric data (weight, height, and waist circumference) of students were collected using a general information questionnaire. To measure anthropometric data, the weight was measured with minimal possible clothing, without shoes, and in a fasting state, and height was measured without shoes in a standing position by a wall-mounted stature meter. Waist circumference was assessed in its thinnest area by a measuring tape with 0.5 cm accuracy while the student was at the end of the normal exhalation. In students with difficult recognition of the thinnest waist area, waist circumference was measured exactly below the last vertebra. All these procedures were instructed to the students, and they personally reported anthropometric data online. In the second part, the students were asked questions about Mukbang-watching frequency in the last week, and their answers were scored on a Likert scale.^19^ In the third part, the Dutch Eating Behavior Questionnaire (DEBQ) was administered under the supervision of an academic and researchers to the students. At first, a pilot study was conducted to further evaluate the questionnaires and the study. In a nonclinical sample of participants in another study, Cronbach’s alpha coefficient for three subscales of eating behavior was 0.94 for restrained eating, 0.97 for emotional eating, and 0.84 for external eating.^20^ In an Iranian study, Cronbach’s alpha coefficient for three subscales of eating behavior was 0.91 for restrained eating, 0.95 for emotional eating, and 0.85 for external eating.^21^ This questionnaire consists of two parts. The first part includes the profiles of subjects and some information on height, weight, weight fluctuations, and the presence or absence of periodic overeating. The second part comprises test questions including 33 questions and three sub-scales measuring emotional, external, and restrained eating behavior. Questions center around people’s eating habits (e.g., Do you have the desire to eat when you are irritated?). The answers are rated on a 5-point degree, with the least and highest degrees receiving the lowest and the greatest scores, respectively (never = 1, seldom = 2, sometimes = 3, often = 4, and very often = 5). The summed score of each subscale forms the raw number of that subscale. To obtain the criterion score, the raw score must be divided by the answered questions of the same scale. If more than one question remains unanswered from each subscale, the score of that scale is not valid and is not included in the calculation.^22^

###  Statistical analysis

 The data were analyzed statistically using SPSS 21. The normality of the data was evaluated by the Kolmogorov-Smirnov test. Qualitative and quantitative variables among various Mukbang watching groups were compared using Fisher’s exact test or chi-square test and the analysis of variance (ANOVA), respectively. Quantitative variables among educational level groups were compared using an independent *t *test. Finally, odds ratios (ORs) and 95% confidence intervals (CIs) among Mukbang watching groups were calculated using the logistic regression test. Data were reported as OR (95% CI), and a *P* value < 0.05 was considered statistically significant.

## Results

 A Mukbang-watching frequency of 60.5% was found among female participants. Mukbang-watching statuses of 12.3% (never), 18.4% (rarely), 19.3%)occasionally), and 10.5%)always(were reported by subjects before writing this article. The mean ± standard deviations of the scores of various eating behaviors in students for total eating behavior, emotional eating, restrained eating, and external eating were 2.66 ± 0.54, 2.36 ± 0.77, 2.88 ± 2.64, and 3.07 ± 0.72, respectively. Table 1 represents the distribution of demographic and anthropometric data, as well as total eating behavior scores among different groups of the studied students based on watching Mukbang. The different Mukbang-watching groups were not significantly different in terms of stature, weight, BMI, waist circumference, educational levels, and total eating behavior scores (*P*> 0.05).

**Table 1:** The distribution of demographic and anthropometric data in different categories of Watching mukbang among the studied students (N = 114)

**Continuous variables**	**Never**		**Rarely**		**Occasionally**		**Always**		***P*** ** value**
	**Mean**	**SD**	**Mean**	**SD**	**Mean**	**SD**	**Mean**	**SD**	
Demographic data									
Age (y)	22.78	3.77	22.23	3.63	21.63	3.63	22.00	3.27	0.686
Eating behavior score	2.64	0.56	2.57	0.46	2.73	0.63	2.90	0.43	0.284
Anthropometric data									
Height (m)	165.22	6.28	165.37	4.72	165.31	4.57	164.41	7.27	0.965
Weight (kg)	59.77	9.90	61.34	14.90	62.54	10.80	62.41	9.74	0.788
BMI (kg/m^2^)	21.90	3.46	22.37	4.93	22.85	3.61	23.15	3.69	0.711
Waist circumference (cm)	69.97	19.71	73.97	18.64	70.63	18.52	75.25	24.22	0.740
**Categorical variables**	**Number**	**%**	**Number**	**%**	**Number**	**%**	**Number**	**%**	***P*** ** value**
Level of education									0.116
Bachelor’s degree	28	33.3	28	33.3	17	20.2	11	13.1	
Masters and PhD	17	56.7	7	23.3	5	16.7	1	3.3	

*Note*. SD: Standard deviation; BMI: Body mass index; PhD: Doctor of philosophy.

Table 2 depicts the distribution of eating behaviors among different groups of the studied female students based on watching Mukbang status. One-way ANOVA revealed no significant relationships between Mukbang-watching frequency and emotional or retrained eating behaviors (*P*> 0.05). However, external eating behavior was significantly related to Mukbang-watching frequency (*P*= 0.0001), so more intensive external eating behavior was noticed with higher Mukbang-watching frequency. Based on Tukey’s post hoc analysis, this significance was also observed between always Mukbang watching and the other Mukbang-watching frequencies (never and always class, *P*= 0.001, rarely and always class, *P* = 0.001, and occasionally and always class, *P* = 0.007).

**Table 2:** Examining the score of eating behaviors in different categories of watching Mukbang among the studied students (N = 114)

	**Never (n=45)**		**Rarely (n=35)**		**Occasionally (n=22)**		**Always (n=12)**		***P*** ** value**
**Eating behavior**	**Mean**	**SD**	**Mean**	**SD**	**Mean**	**SD**	**Mean**	**SD**	
Emotional eating	2.40	0.83	2.27	0.73	2.38	0.87	2.52	0.64	0.780
External eating	3.02	0.76	2.85	0.56	3.08	0.74	3.87	0.40	0.001
Restrained eating	2.59	0.88	2.68	0.87	2.82	0.99	2.44	0.68	0.616

*Note.* SD: Standard deviation.

Table 3 lists the ORs and 95% CIs for eating behaviors among Mukbang-watching groups. Age was the only adjusted confounder in Model 1, and BMI and educational levels were also adjusted besides age in Model 2. The emotional eating chance was 0.88 times higher in people who rarely watch Mukbang than those who never watch Mukbang (they eat emotionally by less than 12%). This chance was 0.81 and 0.54 times greater in people who occasionally and always watch Mukbang, respectively, than those who never watch Mukbang. Moreover, the *P* value was not significant in all raw and adjusted models. The restrained eating chance does not display any special trend. However, it can be claimed that the restrained eating chance is lower and higher in students who always and occasionally watch Mukbang, respectively, than in those who never watch Mukbang. As the *P*-value is significant in the external eating subgroup, the external eating chance is 27% lower in students who rarely watch Mukbang than those who never watch Mukbang. However, this chance is 0.31 and 9.58 times higher in students who occasionally and always watch Mukbang, respectively. Similarly, the external eating chance increases in models adjusted for age, education, and BMI.

**Table 3:** Thestudy of ORs and 95% CIs for eating behaviors in different categories of watching Mukbang among the studied students (N = 114)

**Eating behavior**	**Model 1**		**Model 2**	
	**OR (95% CI)**	***P *** **value**	**OR (95% CI)**	***P *** **value**
Emotional eating				
Never	1.00		1.00	
Rarely	0.90 (0.44, 1.85)	0.774	0.82 (0.39, 1.72)	0.602
Occasionally	0.84 (0.39, 1.83)	0.661	0.79 (0.36, 1.75)	0.568
Always	0.54 (0.20, 1.45)	0.223	0.37 (0.12, 1.14)	0.083
External eating				
Never	1.00		1.00	
Rarely	0.70 (0.33, 1.64)	0.382	0.73 (0.32, 1.62)	0.452
Occasionally	1.24(0.51, 3.01)	0.641	1.23 (0.50, 3.00)	0.623
Always	9.30 (2.57, 33.60)	0.001	12.30 (2.82, 53.65)	0.001
Restrained eating				
Never	1.00		1.00	
Rarely	1.18 (0.69, 2.02)	0.536	1.06 (0.58, 1.93)	0.857
Occasionally	1.56 (0.83, 2.94)	0.162	1.25 (0.51, 3.05)	0.374
Always	0.96 (0.36, 2.56)	0.936	0.64 (0.21, 1.93)	0.434

*Note.* OR: Odds ratio; CI: Confidence interval. Logistic regression analysis. Age was the only adjusted confounder in Model. BMI and educational levels were also adjusted besides age in Model 2.

## Discussion

 The findings of this study indicated a Mukbang-watching frequency of 60.8% among female participants. To our best knowledge, no Iranian study is available on watching Mukbang. A Turkish study reported a Mukbang-watching frequency of 45% among students, 66% of whom were females.^6^ In the present study, based on the obtained findings, no statistically significant relationship was observed between watching Mukbang and people’s age, but in Nam and Jung ’s study in South Korea, a significant relationship was observed between watching Mukbang and people’s age. In this study, the highest and lowest Mukbang-watching frequencies were observed in students aged 30-39 and ≥ 50 years, respectively.^23^ This difference may be due to the fact that more limited age groups were investigated in our study. In the present study, there was no statistically significant difference between watching Mukbang and people’s education.

 Furthermore, one-way ANOVA analysis revealed no significant relationships between Mukbang-watching frequency and emotional or restrained eating behaviors. However, external eating behavior was significantly related to Mukbang-watching frequency, so more intensive external eating behavior was noticed with higher Mukbang-watching frequency. This indicated that an increase in watching Mukbang leads to a higher score in external eating behavior of people who eat more in response to external stimuli probably because they have lost the ability to recognize and behave based on internal hunger signals.^22^ This hypothesis of the positive relationship between watching Mukbang and external eating behavior can be explained using the externality theory related to external stimuli.^13^

 It is believed that external eating is linked to overeating and obesity through various mechanisms. For example, overeating in people with external eating results from their poor understanding of physical hunger and increased sensitivity to external eating signals.^24^ In addition, external eating is reportedly associated with emotional eating, which includes ignoring internal eating signals and eating despite their absence.^25^ This inability to distinguish between emotional excitement and physical hunger results in overeating in response to emotions.^24^ Furthermore, external eating is associated with impulsive personality and personal indiscipline^25,26^ and is reported to have an association with irregular eating behaviors such as overeating in people with a normal weight and overweight with/without eating disorders.^26^

 Anschutz et al reported a positive relationship between external eating and energy, fat, and carbohydrate intakes, whereas restrained eating was negatively correlated with all dependent variables.^27^ Therefore, according to the positive relationship found between external eating and watching Mukbang in our study and according to the results of previous studies in which external eating behavior was associated with increased energy intake, particularly the consumption of fats and carbohydrates,^27^ high Mukbang watching is expected to be associated with increased energy intake, especially the consumption of fats and carbohydrates due to higher external eating behavior in these individuals.

 The findings of the present study are consistent with those of previous studies. In a South Korean study by Nam and Jung, high food delivery and nightly eating frequencies were observed in people who watched Mukbang for more than 14 hours a week and often preferred foods rich in carbohydrates and meats, but more preference for vegetables and fruits was noticed in those who watched Mukbang for less than 7 hours a week.^23^ Schachter believes that overweight and obese people show more reactions to external food signals and have less sensitivity to internal signals of hunger and satiation compared to those with normal weights. However, both psychosomatic and externality theories assume that people with overeating behavior are less aware of internal signals of hunger and satiation. The externality theory also emphasizes that overeaters strongly respond to external food signals such as the view, smell, and flavor of food.^28^ Hence, overweight and obese people are expected to be at more risk for overeating and more energy intake with high Mukbang watching.

 The rise of unhealthy eating behaviors with increasing Mukbang watching has also been reported in studies conducted in other countries. Xu claimed that women who watched Mukbang showed lower satiation levels and were tended to eat more than those who watched non-food content. Overall, all studies by Xu indicated that Mukbang persuaded women who were on a diet to have more desire to consume food.^29^ As reported by Kircaburun et al in Turkey, spectators watch Mukbang for social, fun, and eating purposes or as a compensating escape strategy. Watching Mukbang also seems to have both useful (reducing a sense of solitude and social seclusion and building an online social group) and unfavorable consequences (e.g., changing dietary preferences, eating behaviors, and table behaviors as well as encouraging disordered eating, probable excess, and dependency).^30^ Unlike our results, research by Strand and Gustafsson in Sweden revealed that Mukbang can limit eating and prevent eating in some people. However, this study also demonstrated that Mukbang can increase eating and reduce the feeling of guilt caused by overeating. It is noteworthy that watching Mukbang is not necessarily a helpful or destructive action, but it is useful and detrimental simultaneously.^31^

 Based on our findings in this study, the different Mukbang watching groups were not significantly different in terms of height, weight, BMI, waist circumference, educational levels, and total eating behavior scores. Contrary to the present study, research by Nam and Jung indicated that there are statistically significant differences between weight and BMI with Mukbang-watching frequency, and people’s average weight increased with the rise of watching Mukbang. BMI increased with the increase in watching Mukbang, and men who watched Mukbang more than 7 hours per week were overweight based on BMI. In addition, the highest levels of omitting breakfast, food delivery frequency, and nightly overeating belonged to individuals who watched Mukbang for more than 14 hours a week. This group also showed low interest in health and weekly exercise frequency. According to the results of this study, it seems that with the increase in watching Mukbang, undesirable eating behaviors appeared, which necessitates proper nutrition education to increase the awareness of desirable eating behaviors.^23^

 Our study faced several limitations. First, the cross-sectional design of this study is its main limitation because it does not allow the extraction of causal relationships between watching Mukbang and eating behaviors and anthropometric parameters. Further, even though online studies are common today, due to the coronavirus pandemic and the absence of students in our university, we had to carry out this study online, so the self-reported anthropometric data, especially waist circumference, can be considered another limitation of this study. Despite the limitations, the strengths of this study are worth noting. The value of this study is its uniqueness. To the best of our knowledge, no study in this field has been conducted in Iran so far.

**Highlights**

- There was not a significant relationship between the frequencies of Mukbang watching and emotional or restrained eating behavior.
- There was a significant relationship between external eating behavior and the frequency of watching Mukbang, so with the increase in watching Mukbang, external eating also increased.
- The different Mukbang watching groups were not significantly different in terms of height, weight, body mass index, waist circumference, educational levels, and total eating behavior scores.

## Conclusion

 Based on the obtained findings in this study, no significant relationships were found between Mukbang watching frequency and emotional or restrained eating behaviors. However, external eating behavior was significantly related to Mukbang-watching frequency, so greater intensive external eating behavior was noticed with higher Mukbang-watching frequency. In addition, the different Mukbang watching groups were not significantly different in terms of height, weight, BMI, waist circumference, educational levels, and total eating behavior scores. Therefore, it is necessary to focus on studies concerning eating behaviors, anthropometric parameters, and their association with such programs as Mukbang to prevent related chronic diseases and eating disorders.

## Acknowledgments

 The authors would like to sincerely thank all subjects who participated and consultants who contributed to this study.

## Competing Interests

 The authors declared no conflicts of interest.

## Funding

 None.
